# Loss of *OsHRC* function confers blast resistance without yield penalty in rice

**DOI:** 10.1111/pbi.14061

**Published:** 2023-04-27

**Authors:** Yanpeng Ding, Fuping Zhang, Fangyao Sun, Jilu Liu, Zhenzhen Zhu, Xi He, Guihua Bai, Zhongfu Ni, Qixin Sun, Zhenqi Su

**Affiliations:** ^1^ College of Agronomy and Biotechnology China Agricultural University Beijing China; ^2^ State Key Laboratory for Biology of Plant Diseases and Insect Pests, Institute of Plant Protection Chinese Academy of Agricultural Sciences Beijing China; ^3^ US Department of Agriculture Hard Winter Wheat Genetics Research Unit Manhattan Kansas USA

**Keywords:** rice blast resistance, histidine‐rich calcium‐binding protein, loss‐of‐function mutation, susceptibility gene, wheat FHB

Rice blast, caused by *Magnaporthe oryzae*, is a serious disease in rice (*Oryzae sativa* L.) worldwide. Because the pathogen mutates rapidly, rice blast has become one of the major threats for global rice production (Wang and Valent, 2017). Using host resistance is the most effective method for blast control. However most rice resistance genes are race‐specific (Li *et al*., 2017). Discovering race non‐specific resistance genes will enhance blast resistance in rice cultivars.

*Fusarium graminearum* is a hemibiotrophic fungal pathogen for wheat Fusarium head blight (FHB) and shares a similar lifestyle with *Magnaporthe oryzae* for rice blast. Previously, we have cloned *Fhb1* encoding a histidine‐rich calcium‐binding protein (TaHRC) from wheat for race non‐specific resistance to *Fusarium* species, which has been widely deployed in many wheat cultivars worldwide (Bai *et al*., 2018; Su *et al*., 2019). *TaHRC* serves as a susceptibility factor to promote FHB spread and loss‐of‐function mutation in *TaHRC* confers FHB resistance (Chen *et al*., 2022). *HRC* is highly conservative with the closest *TaHRC* homologue from rice (*OsHRC*), however, the functions of *OsHRC* in rice blast resistance remains to be investigated.

We cloned *OsHRC* (LOC_Os01g03060) from ‘Zhonghua11' (ZH11) and the *OsHRC* DNA sequence is 2390 bp with three exons and two introns. *OsHRC* encodes a putative 33.5 kDa protein of 285‐amino acids and shares 63.5%–64.5% amino acid identity with TaHRC from three wheat subgenomes (A, B and D). The TaHRC and OsHRC proteins were predicted to have similar biological features and both contain a histidine and arginine enriched region without any known functional domain except for a motif of a nuclear localization signal (NLS), indicating the conserved HRC function between rice and wheat. The OsHRC protein was localized to the nucleus in rice leaf protoplasts (Figure 1a). Transactivation activity assay showed that yeast transiently expressing BD‐OsHRC and BD‐TaHRC did not grow in a deficient medium (SD/‐Trp‐His) (Figure 1b), suggesting that both nuclear proteins do not have transactivational activity and share the same biological functions.

**Figure 1:**
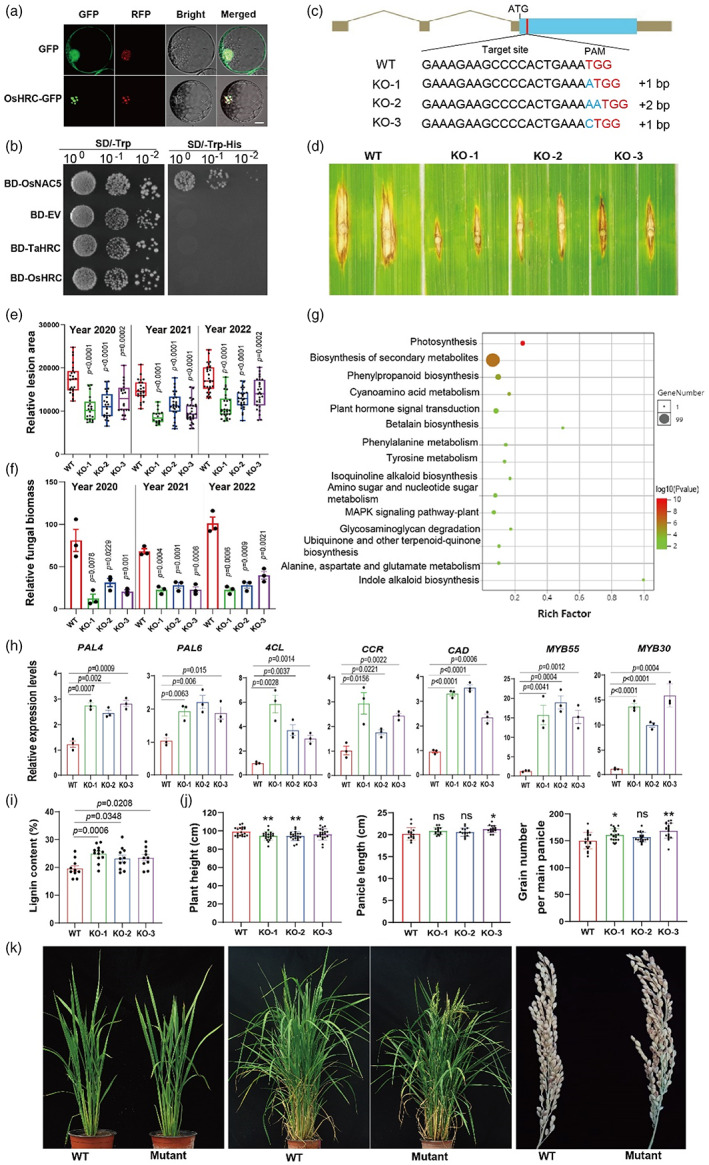
Characterization of *OsHRC* for resistance to rice blast isolate RB22. (a) Subcellular localization of *OsHRC* and *TaHRC* transiently expressed in rice protoplasts. GFP, green fluorescent protein. RFP, red fluorescent proteins, respectively. Scale bar = 5 μm. (b) Transactivation activity assay of *OsHRC* and *TaHRC* in yeast used a rice transcription factor OsNAC5 as a positive control. EV, empty vector. BD, a vector with the DNA binding domain activity. SD/‐Trp, synthetic dropout (SD) medium lacking tryptophan. SD/‐Trp‐His, a SD medium lacking tryptophan and histidine. (c) *OsHRC* gRNA target site showing mutated sequences in the three T1 lines generated from Zhonghua11. CRISPR/Cas9 and gRNA were cloned into a pCBSG032 vector and transformed into Zhonghua11 callus via *Agrobacterium tumefaciens*. Blue letters refer to inserted nucleotides after editing. ATG, start codon. Blue box, ORF. (d–f) Lesions caused by *M. oryzae* on the mutant lines KO‐1, KO‐2 and KO‐3 were significantly smaller with less fungal biomass than in non‐edited Zhonghua11 at 14 days post inoculation (DPI) with RB22. (g) The KEGG analysis of differentially expressed genes in WT and KO‐1 at 14 DPI. (h) Differential expression of the seven lignin‐related genes between three mutants and Zhonghua11 at 14 DPI detected by qRT‐PCR analysis. Data were normalized by *OsActin*. (i) Higher lignin content was observed in the leaves of three mutants than Zhonghua11 at 14 DPI. (j, k) The mutants showed shorter plants, slightly longer panicle and more grain per panicle compared to Zhonghua11. Values are means and standard errors with *n* = 3 (e,f,h), *n* ≥ 10 (i) and *n* ≥ 15 (j).

To verify the function of *OsHRC* on rice blast resistance, we designed a guide RNA to target a 20‐nt sequence in the 59 bp downstream of the translation start codon of *OsHRC* and conducted CRISPR/Cas9‐mediated gene editing to knock out (KO) *OsHRC* in ZH11. Among the five *OsHRC* KO mutants generated, three (KO‐1, KO‐2 and KO‐3) showed premature translation termination due to frameshifts, and were predicted to generate truncated proteins of 39, 236, and 39 amino acids, respectively (Figure 1c). The three mutants and ZH11 were inoculated with RB22, a highly virulent *M. oryzae* strain from China, 60 days after planting. All the three mutants showed significantly smaller lesions and less fungal biomass than their non‐edited controls at 14 days post inoculation (DPI) (Figure 1d–f). To examine whether the resistance is race non‐specific, the three mutants were inoculated with another virulant strain S005 and showed the same level of resistance as to RB22 (Figure S1a,b), demonstrating that the loss‐of‐function of *OsHRC* confers race non‐specific resistance to *M. oryzae*.

To explore the molecular basis of *OsHRC* resistance to rice blast, RNA‐seq was conducted to identify differentially expressed genes (DEGs) between an *OsHRC* KO mutant and ZH11. At 14 DPI with RB22, a total of 1333 DEGs were detected at the fold‐change of 2 and false discovery rate (FDR) < 0.05 using the R package DESeq2. KEGG analysis indicated that the DEGs were mainly enriched in the pathways of photosynthesis, biosynthesis of secondary metabolites, and phenylpropanoid biosynthesis (Figure 1g). Phenylpropanoids play important roles in plant responses to biotic and abiotic stresses (Li *et al*., 2020). Quantitative RT‐PCR results demonstrated that the genes in the phenylpropanoid biosynthesis pathway including genes encoding two phenylalanine ammonia‐lyase (*PAL6*, Os04g0518400; *PAL4*, Os02g0627100), a 4‐coumarate‐CoA ligase (*4CL*, Os01g0901600), a cinnamyl‐CoA reductase (*CCR*, Os02g0180700) and a cinnamyl‐alcohol dehydrogenase (*CAD*, Os04g0229100) were significantly upregulated in the three *OsHRC* mutants compared with ZH11 (Figures 1hand S2a), consistent with Vanholme *et al*. (2019). In addition, the transcription factors, *MYB30* and *MYB55* involved in the regulation of the phenylpropanoid pathway (Li *et al*., 2020) were upregulated in the three *OsHRC* mutants (Figures 1h and S2a). Lignin is one of the most important phenylpropanoid metabolites, and its accumulation in leaf inhibits the penetration of *M. oryzae* (Li *et al*., 2020; Zhou *et al*., 2018). Since all the seven genes are pivotal for the lignin biosynthesis (Vanholme *et al*., 2019), we compared the lignin content between all mutants and the non‐edited control, and found that the leaves of three mutants contained significantly higher lignin content than the control at 14 DPI with the isolate RB22 (Figures 1i and S2b), suggesting that *OsHRC*‐mediated blast resistance is most likely due to the activation of the lignin synthesis pathway after *M. oryzae* infection.

*HRC* is a conserved gene in cereal crops and the loss‐of‐function mutation in *HRC* confers resistance to wheat FHB (Su *et al*., 2019) and maize ear rot without significant penalty on yield (Liu *et al*., 2022). To evaluate effects of *OsHRC* on major rice agronomic traits, we compared heading date, plant height, and grain size between the mutants and WT control in greenhouse experiments and found that the mutants were slightly shorter, but with larger spikes, more spikelets per spike, and earlier heading date than the control (Figure 1j,k). These data indicate that *OsHRC* mutants showed significant improvement in blast resistance without obvious yield reduction in greenhouses, therefore, can be used as a new source of blast resistance in rice breeding.

## Funding

This work was supported by the National Key Research and Development Program of China (2022YFD1201502), Talent Funds of China Agricultural University (2021RC009) and US Wheat and Barley Scab Initiative.

## Author contributions

ZS and GB designed the project and wrote the manuscript. ZS, YD, FZ, ZZ, XH, FS, JL performed experiments. All authors revised and approved the manuscript.

## Conflicts of interest

The authors declare no conflicts of interest.

## Supporting information


**Data S1** Material and methods.
**Figure S1** Comparison of *OsHRC* of rice for responses to the inoculation of *M. oryzae* isolate S005. (a) Lesions caused by *M. oryzae* on the non‐edited control (WT) and three knockout (KO) mutant lines (KO‐1, KO‐2 and KO‐3). (b) Relative lesion areas (means ± standard errors) in the three mutants were significantly smaller than in non‐edited control at 14 days post inoculation (DPI) with the *M. oryzae* isolate S005. (c) Relative fungal biomass (means ± standard errors) in the three mutants was significantly lower than in non‐edited ZH11 control at 14 DPI.
**Figure S2** Comparisons of transcript levels of lignin‐related genes and lignin content in the leaves between ZH11 and the mutants after different time points of inoculation with the *M. oryzae* isolates RB22. (a) Comparisons of seven differentially expressed lignin‐related genes between three mutants and ZH11 (WT control) at 0, 5, 10 and 14 days post inoculation (dpi). Data were normalized using the *OsActin* gene control. Error bars represent the standard error (SE) of three mutants. (b) Comparisons of lignin content between the inoculated and uninoculated leaves of the three mutants and ZH11 (WT control) at 14 DPI with isolate RB22. CK represent uninoculated control. Treatment represents 14 days post inoculation with isolate RB22.
