# Supplementary material for: Loss of OsHRC function confers blast resistance without yield penalty in rice
Source: Plant Biotechnol J. 2023 Apr 27;21(8):1516–8. doi: 10.1111/pbi.14061 (PMC10363764; doi:10.1111/pbi.14061)
Supplement: Supplementary file 1 — Data S1 Material and methods. Figure S1 Comparison of OsHRC of rice for responses to the inoculation of M. oryzae isolate S005. (a) Lesions caused by M. oryzae on the non‐edited control (WT) and three knockout (KO) mutant lines (KO‐1, KO‐2 and KO‐3). (b) Relative lesion areas (means ± standard errors) in the three mutants were significantly smaller than in non‐edited control at 14 days post inoculation (DPI) with the M. oryzae isolate S005. (c) Relative fungal biomass (means ± standard errors) in the three mutants was significantly lower than in non‐edited ZH11 control at 14 DPI. Figure S2 Comparisons of transcript levels of lignin‐related genes and lignin content in the leaves between ZH11 and the mutants after different time points of inoculation with the M. oryzae isolates RB22. (a) Comparisons of seven differentially expressed lignin‐related genes between three mutants and ZH11 (WT control) at 0, 5, 10 and 14 days post inoculation (dpi). Data were normalized using the OsActin gene control. Error bars represent the standard error (SE) of three mutants. (b) Comparisons of lignin content between the inoculated and uninoculated leaves of the three mutants and ZH11 (WT control) at 14 DPI with isolate RB22. CK represent uninoculated control. Treatment represents 14 days post inoculation with isolate RB22. [file PBI-21-1516-s001.docx]

**Supporting information**

**Material and methods**

**Plant materials and growth conditions**

Rice cultivar Zhonghua11 (ZH11) was used to generate *OsHRC* knockout (KO) mutants by gene editing and the edited mutants and non-edited ZH11 control were evaluated for blast resistance and agronomic traits. Rice seeds of both mutants and the ZH11 control were sterilized for 30 min using 2% sodium hypochlorite. After washing for five times with sterile water, the seeds were germinated in small plastic black pots (10 cm × 10 cm) for two weeks. Then the seedlings were transferred into larger pots (170 mm x 190 mm) and cultivated in the greenhouse conditions at 26 ℃ in daytime with 12 h supplemental light and 20 ℃ at night with three replications in China Agricultural University, Beijing, China. Plant height, heading date, spike length and grain number for main panicle per plant were measured from at least 15 plants per genotype prior to harvesting.

**Rice transformation and mutation analysis**

The CRISPR/Cas9 gene editing system was used to knock out *OsHRC* in ZH11. A guide RNA that targets a 20-nt sequence in *OsHRC* was cloned into a *pCBSG032* vector. The generated construct was introduced into the callus of ZH11 via *Agrobacterium tumefaciens*-mediated transformation (Zhang et al., 2020). Mutations was identified by sequencing PCR-products from the target regions of *OsHRC* using a Sanger sequencer.

**Pathogen inoculation and disease resistance assay**

To induce blast disease, *M. oryzae* isolates, RB22 and S005, were delivered to rice leaves using punching inoculation. Spores were produced by growing the isolates on a oatmeal agar medium for 14 days at 25 ℃ in 12 h light and dark conditions. Harvested spores were adjusted to 5 × 10^5^ spores/ml before inoculation. For inoculation, eight-week-old (60 days after planting) rice leaves were gently punched with a mouse ear punch, a 10 μl spore suspension was delivered by a pippette onto the punched site in each leaf, and then both sides of the inoculated site were securely sealed using Scotch tape. At 14 days past inoculation, the picture was taken for each inoculated leaf, and the disease symptoms were evaluated by measuring lesion sizes using the Adobe Photoshop CS6 software. The relative fungal biomass was assayed using DNA-based quantitative PCR (qPCR) as described by [Tao et al. (2021](#_ENREF_3)). Relative fungal growth rate was computed using a cycle threshold (CT) value of *M. oryzae*-specific *MoPot2* gene against the CT value of the rice ubiquitin (*OsUBQ*) gene (Park et al, 2012). Blast phenotyping were conducted in three independent experiments in 2020, 2021 and 2022, respectively.

**Rice protoplast isolation and subcellular localization**

To isolate rice protoplasts, rice seeds were sterilized by immersing them into 75% ethanol for 2 min and then transferring to 2% sodium hypochlorite for 30 min. After washing five times with sterile water, seeds were germinated on a Murashige and Skoog (MS) medium at 26 ℃ under dark. Two-week-oldetiolated rice seedlings were used for protoplast isolation as previously described (Zhang et al., 2020). The sheath and stem of the seedings were cut into 0.3-0.5 mm strips and soaked in 0.6 M mannitol solution for 30 min in the dark with gentle shaking at 80 rpm. Then the strips were filtered using Miracloth nylon mesh and transferred into a cell wall digestion buffer (0.6 M mannitol, 10 mM MES, 1.5% cellulase RS, 0.75% macerozyme R-10, 10 mM CaCl_2_, 0.1% BSA and 50 mM 2-Mercaptoethanol at pH = 5.7) for 5 h with gentle shaking at 80 rpm under dark. After digestion, the strips were washed with a W5 solution (154 mM NaCl, 125 mM CaCl_2_, 5 mM KCl and 2 mM MES at pH 5.7) and filtered with Miracloth nylon mesh. Protoplasts were collected by centrifugation at 100 g for 3 min and washed with the W5 solution for three times, then suspended in an MMg solution (0.6 M mannitol, 15mM MgCl_2_ and 4mM MES at pH = 5.7) at the concentrations of 2×10^5^ to 1×10^6^ cells per ml.

For subcellular localization of the *OsHRC* protein in rice protoplasts, the *OsHRC* coding sequence was inserted into a *pCAMBIA1300-GFP* (green florescent protein) vector to generate *OsHRC-GFP* vector. The *TaHRC* coding sequence and RFP (red florescent protein) tag sequence were inserted together into a *pWMB110* vector to generate the *TaHRC-RFP* vector as the positive control. The *OsHRC-GFP* and *TaHRC-RFP* plasmids were co-transformed into rice protoplasts by PEG-mediated transformation (Zhang et al., 2020). After incubation in the dark for 16 to 20 h at 28 ℃, transformed protoplasts were imaged under a confocal microscope (Zeiss LSM 900).

**Transcriptional activity analysis**

*OsHRC* transactivation was conducted using a Y2H Gold Yeast Hybrid system (Weidi, Shanghai, China). The coding sequences of *OsHRC*, *TaHRC* and *OsNAC5* were cloned into PGBKT7 (Clontech Laboratorise, CA, USA) to fuse to the GAL4 binding domain, respectively. The *OsNAC5* was used as a positive control and empty BD vector was used as a negative control. The constructed vectors were transformed into yeast strain Y2H gold and screened on a selective medium (lacking either Trp or both Trp and His), respectively, to confirm the transcriptional activities of the target genes.

**RNA sequencing and data analysis**

At 14 days after infection with isolate RB22 of *M. oryzae,* rice leaves of a knockout line (KO-1) and Zhonghua11 (WT) were collected for RNA extraction. Total RNA was extracted using TRIzol reagent (Invitrogen, California, USA) following the manufacturer’s protocol. RNA-seq libraries were constructed and sequenced on an Illumina NovaSeq 6000 platform to generate 150 bp paired-end reads. The clean reads after filtration were aligned to the rice reference genome (http://plants.ensembl.org/*Oryza sativa Japonica*/Info/Index) using STAR ([Dobin et al., 2013](#_ENREF_1)). Differentially expressed genes (DEGs) were claimed if log2 (fold change) was ≥ 1 and a *P*-value was < 0.05 using the R package DESeq2. KEGG pathway enrichment analysis was then conducted using KOBAS software ([Mao et al., 2005](#_ENREF_2)).

**Quantitative reverse transcriptase PCR analysis**

Total RNA was extracted from 14-days *M. oryzae-*infected leaves of ZH11 and three knockout rice lines as described for RNA-seq. HiScript II QRT SuperMix (Vazyme, Nanjing, China) was used to generate cDNA. About 1 μg of total RNA and 4× gDNA Wiper mix were incubated at 42 °C for 2 min to remove genome contamination. Then 5× HiScript II QRT SuperMix was added to the reaction mixture and incubated at 25 °C for 10 min, 50 °C for 30 min, and 85 °C for 5 min according to the manufacturer's instructions (Vazyme, Nanjing, China). The first-strand complementary DNA was used for qRT-PCR conducted in a CFX96 Real-time PCR cycler (Bio-Rad) using the SYBR Green PCR Master Mix (Q121, Vazyme, Nanjing China) following the manufacturer's instructions. The *OsActin* was used as the reference gene to normalize expression levels. Gene expression level was analyzed using the ∆∆Ct cycle threshold method in a RQ study software (Thermo Fisher Scientific, Mass, USA) as described previously (Su et al., 2019).

**Lignin content**

At 14 dpi with *M. oryzae,* leaves from non-edited control (WT) and three KO lines were collected, dried to constant weight, pulverized and filtered using a 40-mesh sieve. About 3 mg tissue per sample was collected into a 1.5 mL microcentrifuge tube using the protocol from the manufacturer (Solarbio, Beijing, China). At least 10 leaves from each of the WT and three knockout lines were used to examine lignin content.

**Statistical analysis**

Statistical analysis was performed using software of GraphPad Prism version 9.0. Relative lesion area, fungal biomass, lignin content and agronomy traits between wild type control (ZH11) and the KO mutant lines were compared using two-tailed unpaired t-test method.

**References**

Dobin, A., Davis, C.A., Schlesinger, F., Drenkow, J., Zaleski, C., Jha, S., Batut, P., Chaisson, M. and Gingeras, T.R. (2013) STAR: ultrafast universal RNA-seq aligner. *Bioinformatics* 29, 15-21.

Mao, X., Cai, T., Olyarchuk, J.G. and Wei, L. (2005) Automated genome annotation and pathway identification using the KEGG Orthology (KO) as a controlled vocabulary. *Bioinformatics* 21, 3787-3793.

Park, C. H., Chen, S., Shirsekar, G., Zhou, B., Khang, C. H., Songkumarn, P., et al. (2012). The *Magnaporthe oryzae*effector AvrPiz-t targets the RING E3 ubiquitin ligase APIP6 to suppress pathogen-associated molecular pattern-triggered immunity in rice. Plant Cell 24, 4748–4762.

Tao, H., Shi, X., He, F., Wang, D., Xiao, N., Fang, H., Wang, R., Zhang, F., Wang, M., Li, A., Liu, X., Wang, G.L. and Ning, Y. (2021) Engineering broad-spectrum disease-resistant rice by editing multiple susceptibility genes. *J. Integr. Plant Biol* 63, 1639-1648.

Su, Z., Bernardo, A., Tian, B., Chen, H., Wang, S., Ma, H., Cai, S., Liu, D., Zhang, D., Li, T., Trick, H., St Amand, P., Yu, J., Zhang, Z. and Bai, G. (2019) A deletion mutation in TaHRC confers Fhb1 resistance to Fusarium head blight in wheat. *Nat Genet* **51**, 1099-1105.

Zhang, C., Fang, H., Shi, X., He, F., Wang, R., Fan, J., Bai, P., Wang, J., Park, C.H., Bellizzi, M., Zhou, X., Wang, G.L. and Ning, Y. (2020) A fungal effector and a rice NLR protein have antagonistic effects on a Bowman–Birk trypsin inhibitor. *Plant Biotechnol. J*. 18(11): 2354-2363.

**Supplemental Figures**


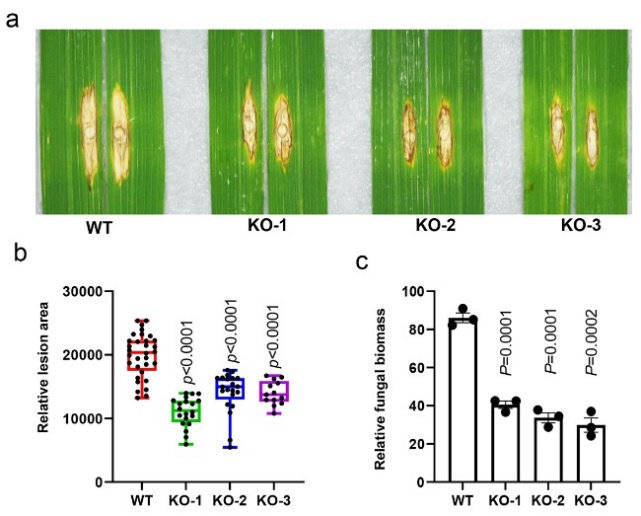


**Supplemental Fig. S1**. Comparison of *OsHRC* of rice for responses to the inoculation of *M. oryzae* isolate S005. (a) Lesions caused by *M. oryzae* on the non-edited control (WT) and three knockout (KO) mutant lines (KO-1, KO-2 and KO-3). (b) Relative lesion areas (means ± standard errors) in the three mutants were significantly smaller than in non-edited control at 14 days post inoculation (DPI) with the *M. oryzae* isolate S005. (c) Relative fungal biomass (means ± standard errors) in the three mutants was significantly lower than in non-edited ZH11 control at 14 DPI.


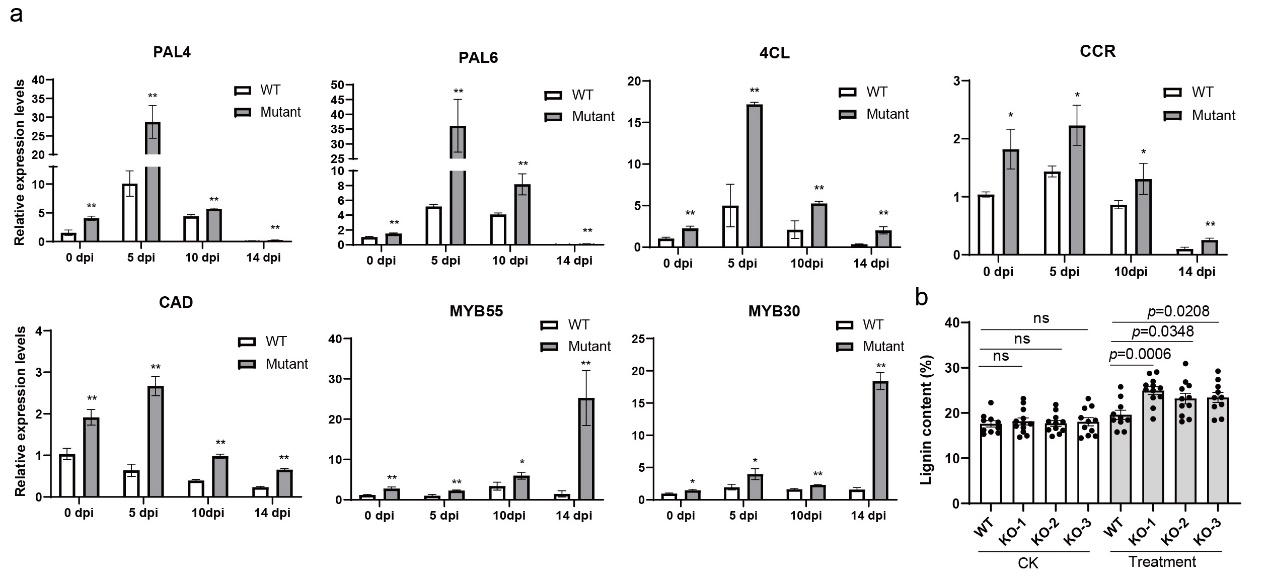


**Supplemental Fig. S2**. Comparisons of transcript levels of lignin-related genes and lignin content in the leaves between ZH11 and the mutants after different time points of inoculation with the *M. oryzae* isolates RB22. (a) Comparisons of seven differentially expressed lignin-related genes between three mutants and ZH11 (WT control) at 0, 5, 10 and 14 days post inoculation (dpi). Data were normalized using the *OsActin* gene control. Error bars represent the standard error (SE) of three mutants. (b) Comparisons of lignin content between the inoculated and uninoculated leaves of the three mutants and ZH11 (WT control) at 14 DPI with isolate RB22. CK represent uninoculated control. Treatment represents 14 days post inoculation with isolate RB22.
